# Ruxolitinib Penetrates Blood Brain Barrier and Reduces the Cytokine Storm in Patients With Haemophagocytic Lymphohistiocytosis

**DOI:** 10.1111/jcmm.71173

**Published:** 2026-05-15

**Authors:** Wenjun Ji, Kai Qing, Jiayi Ren, Kai Xue, Jie Fang, Shishuang Wu

**Affiliations:** ^1^ Department of Pharmacy, Ruijin Hospital, School of Medicine Shanghai Jiaotong University Shanghai China; ^2^ Department of Pharmacy The Affiliated Taizhou People's Hospital of Nanjing Medical University Taizhou China; ^3^ Shanghai Institute of Hematology, State Key Laboratory of Medical Genomics, National Research Center for Translational Medicine at Shanghai, Ruijin Hospital, School of Medicine Shanghai Jiao Tong University Shanghai China

**Keywords:** BBB permeability, CNS‐HLH, ruxolitinib

## Abstract

Haemophagocytic lymphohistiocytosis (HLH), complicated by the involvement of the central nervous system (CNS), contributes to high morbidity and mortality with rapid development and violent cytokine storms in the CNS. Consequently, intrathecal dexamethasone and methotrexate must be administered in a timely manner to treat CNS inflammation. No effective pharmacotherapy targeting cytokine pathways is available to suppress cytokine storms that occur in the CNS. Ruxolitinib, a JAK1/2 inhibitor, has been recommended for the treatment of HLH by multiple guidelines. Conventional studies have reported that ruxolitinib cannot penetrate the blood brain barrier (BBB), thereby impeding the implementation of numerous therapies. Our team previously identified the efficacy of ruxolitinib in patients with CNS‐HLH. Ten patients with secondary HLH (two with CNS involvement and eight without) received ruxolitinib, and BBB permeability was evaluated. Ruxolitinib exhibited BBB penetrability, ranging from 5.31% to 18.08%, suggesting its promising potential in CNS therapy.

## 1 Background

Haemophagocytic lymphohistiocytosis (HLH) is a life‐threatening disorder caused by a severe cytokine storm resulting from abnormal immune regulation (secondary HLH) and genetic mutations (primary HLH). However, owing to its severity and complexity, HLH is frequently misdiagnosed or missed [1, 2]. The disease induces the pathological activation and proliferation of lymphocytes, monocytes and macrophages, ultimately resulting in excessive production of inflammatory cytokines [3]. The median survival of untreated patients with HLH is ≤ 2 months [4, 5]. HLH with central nervous system involvement (CNS‐HLH) is the most serious complication and is triggered by inflammatory cell infiltration into the CNS, which may occur during any course of the disease [6]. Furthermore, 30%–73% of patients with HLH reportedly exhibit CNS symptoms, such as delirium and emotional disturbance [6]. CNS involvement has been identified as an independent risk factor for death in patients with HLH [7], and persistent neurological sequelae remain a critical factor in impairing the quality of life [8]. Timely diagnosis and prompt therapeutic intervention are crucial for alleviating the risk of irreversible CNS injury. Standardised diagnostic criteria and treatment guidelines for CNS‐HLH have not yet been established.

Multiple inflammatory cytokines in the cerebrospinal fluid (CSF), including soluble CD25 (sCD25) [9], IL‐10, IL‐18, IFN‐γ and CXCL9, are reportedly relevant to the occurrence of CNS‐HLH [10]. Thus, therapies targeting inflammatory regulation have been developed as promising approaches for treating CNS‐HLH. As a vital signal pathway of HLH, JAK/STAT participates in the modulation of the levels of various inflammatory factors, including IL‐6, IL‐8, IL‐10, IL‐12, IL‐18, TNF and IFN‐γ [11]; thus, an inhibitor of which is expected to be an attractive therapeutic target. Ruxolitinib, a JAK1/2 inhibitor, has provided satisfactory efficacy in the treatment of HLH, and ruxolitinib has been recommended by multiple guidelines [12, 13]. Ruxolitinib was shown to be effective in a murine model of HIV‐associated neurocognitive disease [14]. However, clinical trials targeting ruxolitinib for the treatment of CNS involvement in patients with HLH have not yet been conducted. The mechanism underlying ruxolitinib‐mediated modulation of CNS inflammatory mediators warrants further clarification.

The effects of anti‐inflammatory drug therapies are dictated by tissue‐specific concentrations. In patients with CNS‐HLH, the anti‐inflammatory efficacy of ruxolitinib in the CSF depends on local exposure. The hydrophilic nature and molecular weight of 404.36 substantially limit CSF penetration. As a promising treatment for CNS‐HLH, the CSF concentration of ruxolitinib may help reveal the blood brain barrier (BBB) permeability and anti‐inflammatory effects.

In this study, we monitored the concentration of ruxolitinib in the CSF of 10 patients with secondary HLH and confirmed that ruxolitinib penetrated the BBB. By tracing the therapy for two secondary CNS‐HLH patients with no familial HLH‐associated mutations or autoimmune disease, a novel notion was developed regarding the BBB permeability of ruxolitinib, which is deemed to play a vital role as an anti‐inflammatory agent. Based on these findings, we postulated the BBB permeability of ruxolitinib in patients with HLH with or without CNS involvement and the inhibition of inflammatory cytokines in the CSF.

## 2 Methods

### 2.1 Study Design and Treatment

In this study, we analysed 17 CSF samples from 10 patients (two with CNS involvement and eight without) with secondary HLH who received ruxolitinib at Ruijin Hospital between February 2025 and August 2025. Patients with HLH were administered oral ruxolitinib at a dose of 20 mg daily, and those with CNS involvement received weekly intrathecal injections. Ruxolitinib dosages were established and optimised by treating physicians according to the patients' conditions, and the therapeutic response to HLH was evaluated biweekly. The diagnosis of CNS‐HLH was confirmed by the presence of neurological symptoms, CSF abnormalities, such as pleocytosis and/or elevated protein levels, or radiographic evidence of parenchymal or meningeal lesions [15]. This observational retrospective study was conducted under natural clinical conditions. The CSF samples tested were surplus samples from patients with normal clinical needs, and no additional trauma or risk was caused to the patients. An Institutional Review Board (http://www.jccro.com/anli/2508.html) waiver was obtained.

### 2.2 Clinical Data Measurement

In the case of a maintained steady state, the trough concentration of ruxolitinib was established. Blood and CSF samples were collected in EDTA tubes 30 min before ruxolitinib administration. CSF inflammation cytokines, including sCD25, IL‐6, IL‐8, IL‐10, IL‐18, IFN‐γ, TNF‐α and IL‐2RA, were collected using the electronic medical record system. The levels of nucleated cells, proteins and glucose in the CSF were detected. Indicators of liver function, ferritin levels and blood biochemistry were evaluated. In addition, imaging examinations and physical profiles, such as temperature, blood pressure, respiratory rate, heart rate and oxygen saturation, were measured.

### 2.3 Methodology Establishment

We established an LC–MS/MS method for the analysis of ruxolitinib concentrations in plasma and CSF samples from patients with HLH. Briefly, sample cleanup was performed using acetonitrile‐mediated protein precipitation. Analyte separation involved gradient elution with mobile phases of 0.1% formic acid in water and acetonitrile. The RUX calibration curves exhibited linearity across 0.100–300 ng/mL.

### 2.4 Statistical Analyses

All data are presented as the mean ± standard deviation (SD). BBB penetration of ruxolitinib was expressed as the CSF/plasma ratio. Pearson's correlation coefficients were used to evaluate the association between ruxolitinib levels in the CSF and plasma. Differences were considered statistically significant at *p* < 0.05.

## 3 Results

### 3.1 Clinical Characteristics of Patients With CNS Involvement

**FIGURE 1:**
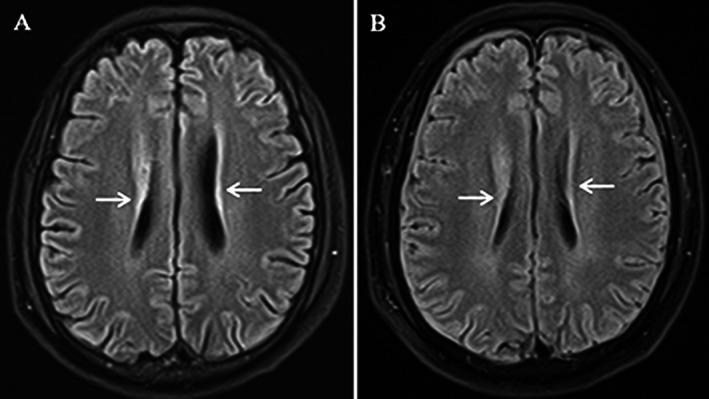
Brain MRI scans at symptom onset (A) and after symptom improvement (B) of Case 1. White arrows point to the patchy hyperintensity.

In this study, we included two patients with secondary HLH with CNS involvement and eight patients without CNS involvement. Two patients (Patients 1 and 2) in this cohort presented with active CNS involvement (Table 1). Patient 1, a 61‐year‐old male with primary bone lymphoma‐triggered HLH, and Patient 2, a 22‐year‐old male with chronic active EBV‐associated HLH, both exhibited significant systemic inflammatory burdens at admission (median ferritin: 5120.5 ng/mL; median sCD25: 1971.5 pg/mL). CNS involvement is characterised by acute neurological symptoms, including tremors, psychomotor agitation and decreased auditory acuity. The diagnosis was confirmed using CSF analysis and neuroimaging. In Patient 1, CSF results during the acute phase showed markedly elevated protein (1915.12 mg/L) and sCD25 (124 pg/mL), with MRI revealing patchy hyperintensity in the lateral ventricular white matter (Figure 1A). Similarly, Patient 2 exhibited elevated levels of CSF cytokines and sCD25 (70 pg/mL). The pharmacokinetic analysis of active neuroinflammation revealed significant BBB penetration by ruxolitinib. In Patient 1, BBB permeability reached 15.98% (CSF concentration: 5.05 ng/mL), whereas in Patient 2, it reached a peak permeability of 14.04% (CSF concentration: 4.01 ng/mL) during disease progression. Notably, as systemic and CNS inflammation subsided in Patient 1 after 8 days of ruxolitinib‐based therapy, BBB permeability decreased to 9.15%, correlating with the resolution of white matter lesions on MRI (Figure 1B) and clinical stabilisation. These data suggested that the degree of ruxolitinib penetration was dynamically regulated by the inflammatory status of the CNS (detailed longitudinal clinical courses are provided in the Supporting Information).

**TABLE 1:** Lab results of CSF for Patients 1 and 2.

Time	Patient 1		Patient 2	
	CNS involvement	CNS improved	CNS involvement	CNS improved
Ruxolitinib in CSF (ng/mL)	5.05	3.51	1.91	2.83
Ruxolitinib in plasma (ng/mL)	31.61	38.34	19.56	36.65
BBB penetration of ruxolitinib (%)	15.98	9.15	9.76	7.72
CSF sCD25	124	25	70	37
CSF protein	1915.12	631.35	645.36	637.16
CSF glucose	3.46	2.5	2.41	2.47
CSF nucleated cells	35 × 106	17 × 106	12 × 106	6 × 106
CSF IL‐6 (< 5.4 pg/mL)	33.2	27.2	4.2	21.3
CSF IL‐8 (< 20.6 pg/mL)	985.1	332.2	341.4	265.3
CSF IL‐18 (< 87.7 pg/mL)	75.44	18.37	40.02	18.69
CSF IL‐2RA (< 610.1 pg/mL)	765.55	132.87	1263.56	326.8
CSF TNF‐a (< 16.5 pg/mL)	160.9	7.5	—	—
CSF IFNr (< 23.1 pg/mL)	26.4	9	—	—
CSF IL‐10 (< 12.9 pg/mL)	23.9	< 2.4	< 2.4	< 2.4

### 3.2 Comparative Analysis of Clinical Characteristics Between CNS‐Involved and Non‐CNS Groups

To evaluate the clinical landscape associated with CNS involvement in HLH, we performed a comparative analysis between patients with (*n* = 2) and without (*n* = 8) CNS involvement (Tables 2 and 3). The median age of the CNS involved group was 41.5 years, which was lower than the 54.5 years observed in the non‐CNS group. This trend may suggest a higher susceptibility to neurological invasion in younger patients or in those with specific genetic predispositions. Notably, both cohorts presented with persistently high fever upon admission, with median temperatures exceeding 38.5°C, underscoring the systemic hyperinflammatory state in HLH, regardless of CNS status. To ensure the validity of the subsequent pharmacokinetic comparisons, a uniform therapeutic approach was used. Most patients in both groups received ruxolitinib‐based combination therapy (e.g., ruxolitinib + DEP), providing a consistent pharmacological background for assessing the BBB penetration of ruxolitinib. Quantitative analysis of core biomarkers revealed that the CNS‐involved group maintained consistently high systemic inflammatory loads, with median ferritin levels of 5120.5 ng/mL and sCD25 levels of 1971.5 pg/mL. In contrast, while the non‐CNS group exhibited lower median values (ferritin: 4074.34 ng/mL; sCD25:1734.36 pg/mL), it included significant outliers, such as Patient 3 (ferritin: 15,000 ng/mL). These findings imply that CNS involvement is associated with a persistent and difficult‐to‐control pattern of systemic immune activation.

**TABLE 2:** Baseline demographic and clinical characteristics.

Parameter (median)	CNS involvement group (*n* = 2)	Non‐CNS involvement group (*n* = 8)	Total patients (*n* = 10)
Age (years)	41.5	54.5	53
Temp (°C)	39.05	38.65	38.85
ANC (×10^9^/L)	2.655	2.035	2.15
Haemoglobin (Hb) (g/L)	95.5	70.5	74
Platelets (PLT) (×10^9^/L)	160	48.5	57
Triglycerides (TG) (mmol/L)	2.35	1.49	1.52
Fibrinogen (FBG) (g/L)	4.4	1.4	1.4
Ferritin (ng/mL)	5120.5	4074.34	4679.68
sCD25 (pg/mL)	1971.5	1734.36	1762.71
Splenomegaly (%)	100%	87.50%	90%
CSF/Plasma ratio	15.01%	12.82%	11.33%
NK‐cell activity	28.28%	12.36%	12.36%

**TABLE 3:** Baseline demographic and clinical characteristics of 10 patients.

Parameters	1	2	3	4	5	6	7	8	9	10
CNS involvement	Y	Y	N	N	N	N	N	N	N	N
Gentle	M	M	M	F	M	M	M	M	M	F
Age	22	61	21	60	59	52	19	56	60	53
Therapeutic regimen (28d)	Ruxolitinib + DEP	Ruxolitinib + HLH‐94	Ruxolitinib + DEP	Ruxolitinib + DEP	Ruxolitinib + DEP	Ruxolitinib + HLH‐94	Ruxolitinib + DEP	Ruxolitinib + DEP	Ruxolitinib + DEP	Ruxolitinib + Dexamethasone
Temperature (°C)	39	39.1	39.4	37	38.6	39.2	39.4	38.7	37	37.4
ANC (×10^9^/L)	0.46	4.85	3.38	0.86	2.32	1.5	1.92	2.49	2.15	1.81
Hb (g/L)	105	86	61	74	59	54	116	113	67	111
PLT (×10^9^/L)	261	59	14	106	32	44	27	53	57	168
TG (μmol/L)	2.47	2.23	2.06	1.84	1.45	1.1	1.67	1.06	1.52	1.46
FBG (g/L)	1.6	7.2	1.4	1.4	1.1	3.5	1.3	2.4	1.4	1.88
Ferritin (ng/mL)	5134	5107	15,000	3469	15,000	1351	155,787	1187.5	1949	4679.68
sCD25 (pg/mL)	3713	230	16,700	1331	1706	6210	957.25	15	2386	1762.71
Splenomegaly	Y	Y	Y	Y	Y	Y	N	Y	Y	Y

### 3.3 Profiles of Ruxolitinib BBB Permeability in Secondary HLH With or Without CNS Involvement

**FIGURE 2:**
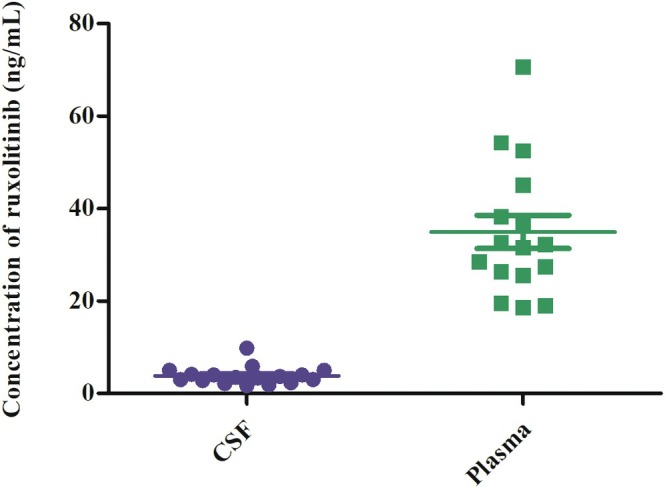
Ruxolitinib levels in CSF and plasma of patients with HLH (samples = 17, *n* = 10).

**FIGURE 3:**
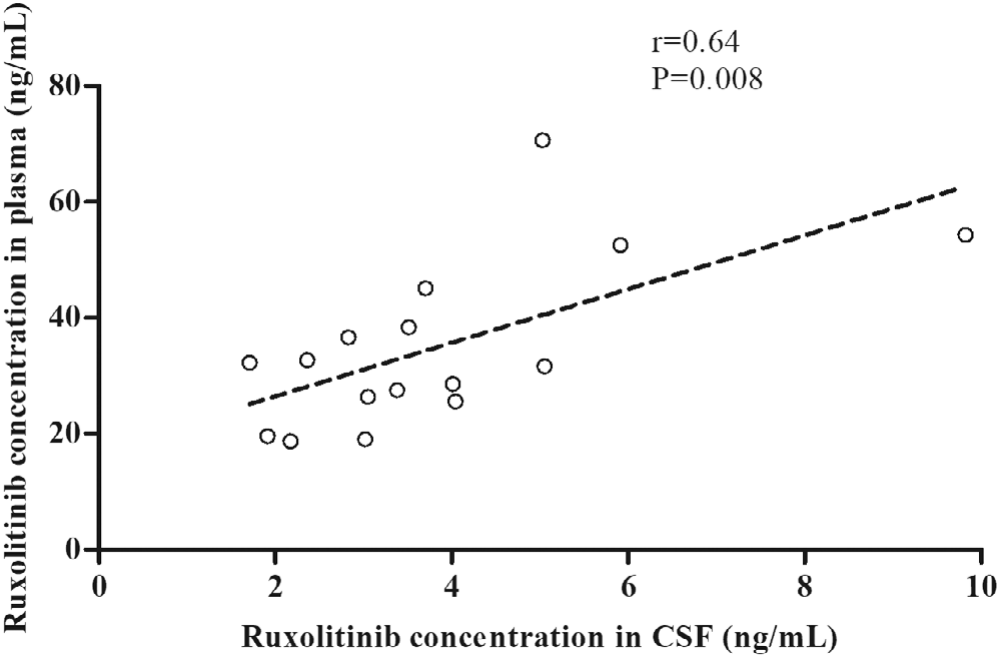
Correlation between ruxolitinib concentration in CSF and plasma in patients with secondary HLH (samples = 17, *n* = 10); Pearson's correlation coefficients were employed to analyse the correlation of ruxolitinib concentration in CSF and plasma (*r* = 0.637, *p* = 0.008).

To clarify the BBB permeability of ruxolitinib, we enrolled eight additional patients with secondary HLH without CNS involvement. All patients with HLH received oral ruxolitinib. CSF and plasma levels of ruxolitinib were evaluated, as shown in Figure 2. LC–MS/MS analysis of ruxolitinib facilitated the assessment of BBB permeability. The value of ruxolitinib *C*_min_ was 3.90 ± 0.44 ng/mL in CSF and 35.23 ± 3.39 ng/mL in plasma. BBB permeability of ruxolitinib was 11.33% ± 0.89% (between 5.31% and 18.08%) (Table 4). Furthermore, Pearson's correlation coefficients were used to analyse the correlation between ruxolitinib concentrations in CSF and plasma. A significant correlation was observed between these variables (*r* = 0.637, *p* = 0.008) (Figure 3). Although systemic plasma concentrations were comparable, the median BBB penetration ratio was higher in the CNS‐involved group (15.01% vs. 12.83%). These data strongly support the mechanistic hypothesis that severe localised neuroinflammation in patients with CNS‐HLH compromises BBB integrity, thereby facilitating the more efficient transit of ruxolitinib into the brain parenchyma. In the CNS group, the value of ruxolitinib *C*_min_ was 4.53 ± 0.52 ng/mL in CSF and 30.09 ± 1.52 ng/mL in plasma. BBB permeability of ruxolitinib was 15.01% ± 0.01% (between 14.04% and 15.98%). In the non‐CNS group, the value of ruxolitinib *C*_min_ was 4.06 ± 1.03 ng/mL in CSF and 30.82 ± 4.78 ng/mL in plasma. BBB permeability of ruxolitinib was 12.83% ± 1.59% (between 5.31% and 18.08%). Crucially, the median CSF concentration of 4.53 ng/mL of ruxolitinib in the CNS group reached the therapeutic threshold required to inhibit JAK/STAT pathway phosphorylation in vitro. This provides a pharmacological explanation for the rapid resolution of neurological symptoms, such as seizures and impaired consciousness, observed in Patients 1 and 2, despite their critical systemic condition.

**TABLE 4:** The BBB penetration of ruxolitinib.

Sample	BBB penetration of ruxolitinib (%)
1	9.76
2	7.72
3	14.04
4	18.08
5	15.84
6	11.59
7	15.98
8	7.22
9	12.30
10	9.15
11	8.20
12	7.12
13	11.25
14	5.31
15	15.80
16	11.56
17	11.63
Mean ± SD	11.33 ± 0.89

## 4 Discussion

Treatment of CNS‐HLH is arduous and time‐consuming for several reasons. The CNS is an immune‐specialised sanctuary site characterised by a lack of resident leukocytes within the brain parenchyma and CSF. Second, the intense cytokine storm that occurs in the CNS results in severe brain tissue injury. Third, the BBB decreases the passive diffusion and targeted delivery of medicine into the CNS. Successful management of CNS‐HLH is contingent on the BBB permeability of the treatment and its efficacy in inhibiting inflammatory cytokines. Ruxolitinib, a JAK1/2 inhibitor, is a promising therapeutic option. Currently, it is recommended for HLH because of its ability to suppress inflammatory factors [16]. In addition, ruxolitinib has been detected in the brains of murine models with HIV encephalitis. However, there is no relevant clinical research on the effect of ruxolitinib in alleviating the CSF cytokine storm in CNS‐HLH. Our study reported two cases of secondary HLH accompanied by CNS involvement and explored the BBB permeability and anti‐inflammatory effects of ruxolitinib.

In this study, inconsistent with the manufacturer's instructions, we reported that ruxolitinib could penetrate the BBB based on the evaluation findings from 10 patients with HLH. Our analysis revealed that the value of ruxolitinib *C*_min_ was 3.90 ± 0.44 ng/mL in CSF, 35.23 ± 3.39 ng/mL in the plasma and 11.33% ± 0.89% in BBB penetration (between 5.31% and 18.08%), which first demonstrated the BBB permeability of ruxolitinib and provided evidence for the composition of the CNS‐HLH regimen. Consistent with these results, Haile et al. reported the therapeutic effects of orally administered ruxolitinib in mice with HIV encephalitis by suppressing the infiltration of monocytes/macrophages, which benefited from its BBB permeability [12].

However, the pathophysiology of CNS‐HLH remains poorly understood. The proposed mechanisms include the transmigration of inflammatory cytokines across the BBB or direct induction by primary or acquired pathological triggers [17]. BBB dysfunction is a pathological feature of several neurological diseases. Ma et al. described brain MRI abnormalities in 71.2% of paediatric patients with CNS‐HLH, who were identified to have diffuse white matter lesions accompanied by parenchymal atrophy [18]. Unusual brain MRI findings were confirmed in Patient 1, who exhibited behavioural and linguistic abnormalities with patchy hyperintensity in the white matter of the lateral ventricle.

Cytokine storm in HLH has been implicated in disrupting the BBB and facilitating the influx of inflammatory cytokines into the brain. This process induces neuroinflammation and is associated with a marked elevation in CSF sCD25 levels. A fulminant cytokine storm, such as elevated IL‐10 or sCD25 levels, is characteristic of HLH [19]. Meanwhile, significant elevation of IL‐6, IL‐8, IL‐18, IFN‐γ and TNF‐α was observed in both primary and secondary HLH, which were mainly triggered via JAK/STAT [20]. Some reports have described that inflammatory cytokines in the CSF help monitor the activity of CNS‐HLH and disease diagnosis [7]. For paediatric HLH patients, the IFN‐γ, sCD25, IL‐10 and IL‐18 levels of CSF were demonstrated to be significantly elevated in the case of CNS involvement compared with those without [10]. In line with the above results, CSF levels of sCD25, IL‐10, IL‐8, IL‐18, TNFα, IL‐2RA and IFN‐γ were reportedly higher in patients with HLH during CNS involvement. Furthermore, our study confirmed that ruxolitinib could improve the symptoms of CNS‐HLH and decrease the CSF levels of sCD25, IL‐10, IL‐8, IL‐18, TNFα, IL‐2RA and IFN‐γ. Notably, IL‐6 levels have not been shown to play a vital role in CNS‐HLH.

Multiple variables influence the BBB penetration of ruxolitinib, including plasma concentrations and the degree of BBB compromise. Systemic administration depends on BBB disruption when patients with HLH develop CNS involvement. Evidence indicates that inflammatory cytokines may impair the BBB and enhance its penetration [19], which may underlie the enhanced BBB penetration of ruxolitinib in HLH. However, this evidence cannot predict whether ruxolitinib crosses the BBB in HLH. Therefore, clinical research is urgently needed to validate the BBB penetrability of ruxolitinib.

To the best of our knowledge, this is the first study to compare CSF ruxolitinib concentrations in involved and uninvolved CNS tissues in patients with HLH. Haile et al. demonstrated that ruxolitinib decreases the infiltration of monocytes/macrophages in vitro and crosses the BBB to restrain astrocyte aggregation in the brains of HIVE mice [12]. This challenges the assertion on product labels that ruxolitinib lacks BBB penetration, which is derived exclusively from animal models. In line with the above findings, we report two cases focusing on the BBB penetration of ruxolitinib in CNS‐HLH. In our study, Patient 1 exhibited BBB permeability of ruxolitinib with CNS involvement of approximately 15.98%, whereas that without CNS involvement was only 9.15%. Similarly, Patient 2 was reported to have a higher BBB permeability of 14.04% for ruxolitinib in the impaired CNS compared with 7.72% in the intact regions of the CNS. Based on these findings, we postulated that the concentration of ruxolitinib may be higher in patients with HLH with CNS involvement.

Currently, intrathecal administration of dexamethasone and methotrexate is recommended for patients with CNS‐HLH. However, HLH is commonly associated with thrombocytopenia (platelets < 100 × 10^9^/L), and intrathecal administration is a suboptimal treatment option. Ruxolitinib is a promising therapy with BBB penetration ranging from 5.31% to 18.08%, as established in our study. It has been postulated that an increase in the dose of ruxolitinib augments drug concentration in the CSF, leading to improved treatment outcomes. Similarly, penicillin, a routine drug of bacterial meningitis in children, lacks BBB penetrance, exhibiting 5%–30% penetration in the damaged CNS compared with 1%–3% in intact regions of the CNS. To address this disadvantage, the dose of penicillin was increased from 50,000–100,000 to 300,000–400,000 U/kg/day to enhance therapeutic efficacy [21].

However, this study has some notable limitations. First, the sample size was small because HLH is a rare haematopathy. Second, because this was a retrospective study, prospective research is urgently needed to validate the BBB penetrability of ruxolitinib. Third, determining the optimal dose of ruxolitinib is essential for the treatment of CNS‐HLH.

In conclusion, BBB penetration of ruxolitinib makes it a candidate for the treatment of CNS‐HLH. Moreover, ruxolitinib may play a vital role in suppressing cytokine storms that occur in the CNS. Based on these findings, oral ruxolitinib is a promising therapy for CNS‐HLH in clinical scenarios in which intrathecal treatment is inconvenient or unavailable.

## Author Contributions

**Wenjun Ji:** data curation, investigation, visualization, writing – original draft. **Jie Fang:** writing – review and editing. **Jiayi Ren:** methodology. **Shishuang Wu:** software, validation, formal analysis, project administration, resources. **Kai Qing:** conceptualization, funding acquisition. **Kai Xue:** supervision, writing – review and editing.

## Funding

This work was supported by the Natural Science Foundation of Tibet Autonomous Region (XZ2024ZR‐ZY048(Z)E T) and the Natural Science Foundation of Shigatse (RKZ2024ZR‐003(Z)).

## Conflicts of Interest

The authors declare no conflicts of interest.

## Supporting information


**Data S1:** Cases with CNS involvement.

## Data Availability

The data that support the findings of this study are available on request from the corresponding author. The data are not publicly available due to privacy or ethical restrictions.
