# Supplementary material for: Ruxolitinib Penetrates Blood Brain Barrier and Reduces the Cytokine Storm in Patients With Haemophagocytic Lymphohistiocytosis
Source: J Cell Mol Med. 2026 May 15;30(10):e71173. doi: 10.1111/jcmm.71173 (PMC13177334; doi:10.1111/jcmm.71173)
Supplement: Supplementary file 1 — Data S1: Cases with CNS involvement. [file JCMM-30-e71173-s001.docx]

Supplementary Cases with CNS involvement

Supplementary Note 1: Detailed Clinical Course of Case 1

Patient 1 (61-year-old male) presented with a 1-month history of emaciation and fever. At admission, the GCS score was 15. Initial lab results included Ferritin 3391 ng/L, sCD25 4037 pg/mL, Hemoglobin 70 g/L, and Platelets 82 × 10^9/L. Following diagnosis of primary bone lymphoma-related HLH, treatment with oral ruxolitinib (10 mg BID) and dexamethasone (2 mg QD) was started. On Day 2, the patient developed tremors and slow speech. MRI showed patchy white matter hyperintensity. The condition worsened to psychomotor agitation and pressured speech. Lumbar puncture revealed nucleated cells at 35 × 10^6/L and glucose at 3.46 mmol/L. After 8 days of combined ruxolitinib and intrathecal therapy (MTX/Dex), the patient became afebrile, and mental symptoms significantly improved. The patient eventually achieved stability as a bridge to allo-HSCT.

Supplementary Note 2: Detailed Clinical Course of Case 2

Patient 2 (22-year-old male) presented with CAEBV-triggered HLH, characterized by high fever (40℃) and pancytopenia (WBC 1.97 × 10^9/L, Hgb 103 g/L, PLT 147 × 10^9/L). Bone marrow showed active hemophagocytosis. Initial treatment included ruxolitinib (10 mg BID) and dexamethasone. Neurological symptoms began with dizziness and hearing loss. Early CSF analysis showed sCD25 at 70 pg/mL and protein at 645.36 mg/L. Although systemic markers improved by Day 4 (PLT rose to 261 × 10^9/L), the patient’s neurological symptoms later progressed alongside an increase in BBB permeability to 14.04%. Despite salvage therapy with the L-DEP regimen, the patient’s prognosis remained dismal due to the underlying CAEBV infection; the patient passed away following transfer for allo-HSCT.
